# Neighbourhood deprivation and cardiometabolic outcomes in the UK Biobank: differences by sex and ethnicity

**DOI:** 10.1136/openhrt-2025-003225

**Published:** 2025-05-27

**Authors:** Kosuke Tamura, Yangyang Deng, Breanna Rogers, Mohammad Moniruzzaman, Ram Jagannathan, Lu Hu, Katsuyuki Miura, Véronique L Roger, Leonardo Mariño-Ramírez

**Affiliations:** 1 Socio-Spatial Determinants of Health (SSDH) Laboratory, Population and Community Health Sciences Branch, Division of Intramural Research, National Institute on Minority Health and Health Disparities, National Institutes of Health, Bethesda, Maryland, USA; 2 Emory Global Diabetes Research Center, Woodruff Health Sciences Center, Emory University, Atlanta, Georgia, USA; 3 Department of Populataion Health, NYU Grossman School of Medicine, New York, New York, USA; 4 NCD Epidemiology Research Center, Shiga University of Medical Science, Otsu, Shiga, Japan; 5 Heart Disease Phenomics Laboratory, Epidemiology and Community Health Branch, Division of Intramural Research, National Heart, Lung, and Blood Institute, National Institutes of Health, Bethesda, Maryland, USA; 6 Division of Intramural Research, National Institute on Minority Health and Health Disparities, National Institutes of Health, Rockville, Maryland, USA

**Keywords:** EPIDEMIOLOGY, RISK FACTORS, Death, Sudden, Cardiac

## Abstract

**Objective:**

To examine the associations of deprived neighbourhoods with all-cause mortality and incident cardiovascular disease (CVD) and to investigate whether these associations were independently and concurrently stratified by sex and ethnicity.

**Methods:**

Data came from the UK Biobank, a prospective cohort study of over 500 000 participants aged 22–69 across the UK between 2006 and 2010. The follow-up time was calculated from each participant’s enrolment at baseline until the first occurrence of a diagnosis of each death, incident or the censor date (31 December 2020). All-cause mortality, incident total CVD, ischaemic heart disease (IHD) and cerebrovascular disease (CeVD) were the outcomes defined based on the International Classification of Diseases. Deprived neighbourhoods were categorised into four groups: least deprived (referent), somewhat deprived, deprived, and most deprived neighbourhoods. Cox proportional hazards models were used to examine associations of deprived neighbourhoods with each outcome. Analyses were stratified by sex and ethnicity separately and simultaneously.

**Results:**

A total of 261 954 participants were included. Participants had a mean follow-up of 14.3 years for all-cause mortality (3 745 307 person-years, 9933 deaths) and 12.7 years for total CVD incidence (3 321 619 person-years, 64 748 events). Those in the most deprived neighbourhoods (compared with the least) had a 31%, 13%, 15% and 34% greater risk of all-cause mortality, incident total CVD, IHD and CeVD, respectively. Patterns of associations were somewhat similar by sex, yet varied by ethnicity. The overall results were consistent with the white cohort but not for the other cohorts.

**Conclusions:**

This study indicated that individuals living in highly deprived neighbourhoods may have an elevated risk of all-cause mortality and incident CVD, particularly among the white cohort but not other cohorts. Future research should focus on efforts to invest in deprived areas to alleviate the burden of all-cause mortality and CVD incidence.

**WHAT IS ALREADY KNOWN ON THIS TOPIC**

- Previous studies have shown that individuals living in deprived or disadvantaged neighbourhoods, compared with those in wealthy or advantaged neighbourhoods, had a greater risk of all-cause mortality and incident cardiovascular diseases (CVD).
- However, there were few studies with stratified analyses by sex and racial and/or ethnic groups, and the findings are mixed.

**WHAT THIS STUDY ADDS**

- This study investigated whether deprived neighbourhoods were associated with increased risk of all-cause mortality, incident total CVD, ischaemic heart disease (IHD), and cerebrovascular disease (CeVD).
- Analyses were stratified by sex and ethnic background independently and simultaneously in the UK Biobank cohort. Living in deprived neighbourhoods increased the risk of all-cause mortality and incident total CVD, IHD and CeVD for both sexes.
- Despite these established links, these associations were observed only in the white cohort and were not evident among other ethnic backgrounds.

**HOW THIS STUDY MIGHT AFFECT RESEARCH, PRACTICE OR POLICY**

- Overall, living in deprived neighbourhoods has adverse effects on all-cause mortality and incident CVD among UK Biobank participants, particularly within the white female and male cohorts.
- Further research should consider oversampling black and Asian cohorts and emphasise addressing the disadvantages of these neighbourhoods to mitigate the burden of all-cause mortality and CVD incidence.

## Introduction

 Cardiovascular disease (CVD), including coronary heart disease, stroke and other cardiovascular conditions, continues to be the leading cause of mortality and morbidity worldwide.^1^ Despite the decline in CVD mortality, CVD remained the second most common cause of death in 2020, affecting 7.6 million UK people with CVD-related diseases.^2^ Efforts to diminish disparities in longevity and CVD outcomes underscored social determinants of health.^3^ Conventional CVD epidemiology lacks consideration of social and environmental contexts.^4^

There has been increasing emphasis on studying the influence of the built environment^5 6^ or neighbourhood socioeconomic deprivation^7 8^ on CVD risk.^9^ To address disparities in mortality and CVD outcomes, research investigated the associations of neighbourhood deprivation with mortality and CVD, with mixed findings by sex,^10^ which remained unclear. Further, the associations of deprived neighbourhoods with mortality and CVD outcomes may differ by racial and/or ethnic groups.

Nonetheless, research investigating sex and ethnicity-specific associations of neighbourhood deprivation with all-cause mortality and CVD outcomes may be incomplete. Thus, the objectives of this study were to (1) examine how living in deprived neighbourhoods would increase the risk of all-cause mortality and incident CVD outcomes and (2) vary such associations by sex and ethnic groups separately and concurrently in the UK Biobank cohort.

## Methods

### Study participants and design

**Figure 1:**
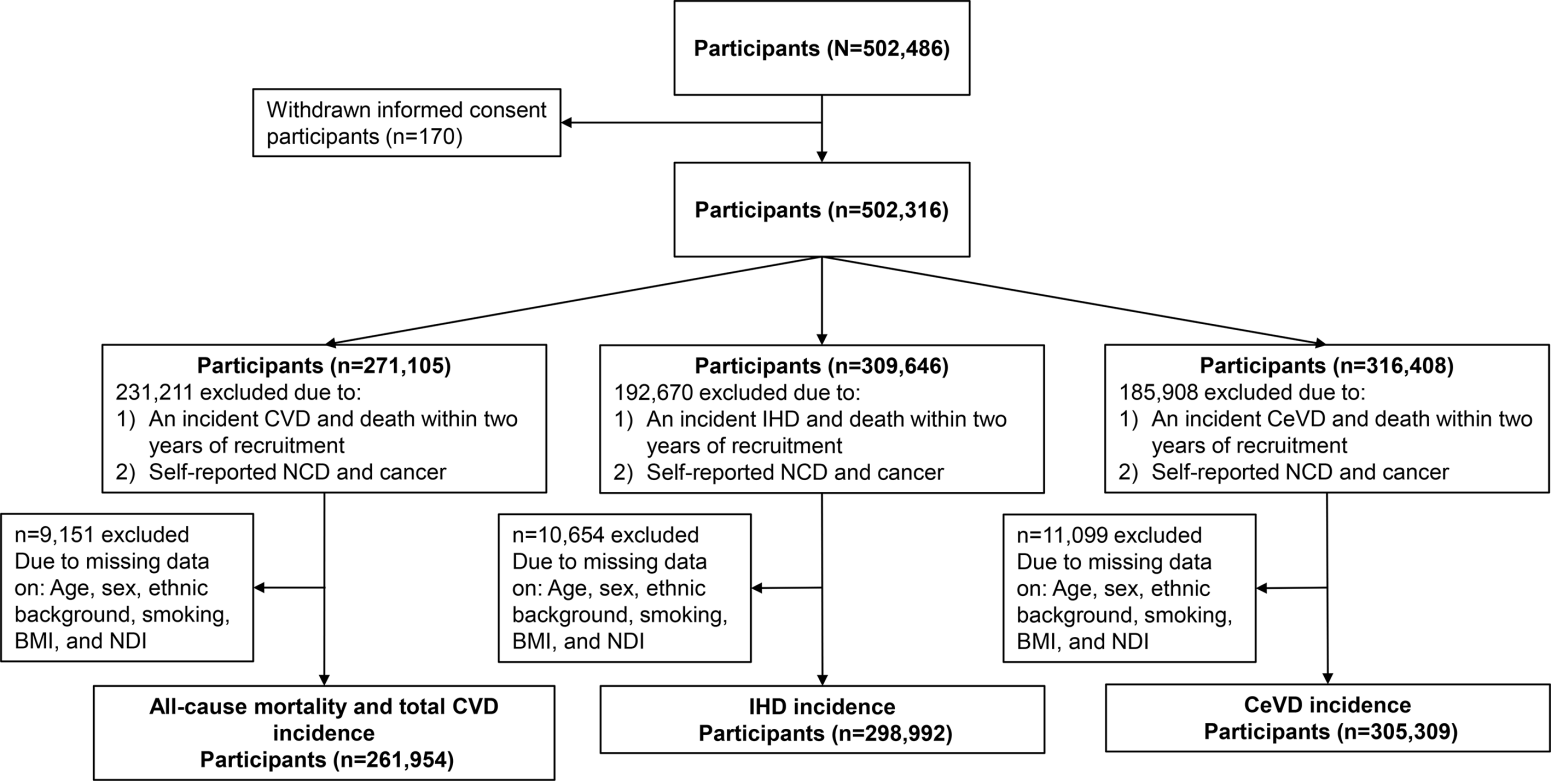
Flow chart for the excluded UK Biobank participants and respective study samples. NCD included depression, bipolar disorder, schizophrenia, alcohol problems, substance abuse, eating disorder, cognitive impairment and dementia, Parkinson’s disease, chronic pain syndrome, chronic obstructive pulmonary disease, chronic pain syndrome, chronic asthma, chronic liver disease, stroke, transient ischaemic attack, diabetes, inflammatory, cardiomyopathy, pulmonary embolism, deep vein thrombosis, arthritis and cancer. BMI, body mass index; CeVD, cerebrovascular disease; CVD, cardiovascular disease; IHD, ischaemic heart disease; NCD, non-communicable disease; NDI, Neighbourhood Deprivation Index.

The UK Biobank, a prospective population-based cohort study, recruited 502 486 participants aged 22–69 years between 2006 and 2010 in 22 assessment centres across the UK. Participants completed questionnaires and provided biological samples.^11^ The follow-up time for each outcome was calculated from each participant’s enrolment at baseline until the first occurrence of a diagnosis of each outcome, death or the censor date (31 December 2020). Of 502 486 participants, 170 did not provide informed consent (figure 1). Analyses were restricted to participants who did not have an incidence (CVD event or death) within 2 years of recruitment and any self-reported non-communicable diseases^12^ and cancer at baseline to minimise the reverse causality bias (231 211 excluded for all-cause mortality and total CVD incidence, 192 670 excluded for ischaemic heart disease (IHD) and 185 908 excluded for cerebrovascular disease (CeVD)). Further, participants were removed due to missing demographic, health-related factors, and the exposure variable (9151 excluded for all-cause mortality and total CVD, 10 654 excluded for IHD and 11 099 for CeVD), resulting in the total analytical samples of n=261 954 for all-cause mortality and total CVD incidence, n=298 992 for IHD and n=305 309 for CeVD. All participants provided written informed consent. This study followed the Strengthening the Reporting of Observational Studies in Epidemiology reporting guideline.

### Outcome ascertainment

The study included four outcomes based on the definition with the three-digit code of the International Classification of Diseases 10th Revision: all-cause mortality, total CVD (I05–I15, I20–I28, I30–I89),^12^ IHD (I20–I25)^13^ and CeVD (I60–I69).^13^

### Exposures

The Neighbourhood Deprivation Index (NDI) was defined as an area-level proximity measure for neighbourhood socioeconomic status (SES). It was computed based on national census data.^14^ NDI was created for all participants when they were enrolled.^15^ Each area had an average household of 125, with 309 individuals. A higher score indicated a more deprived neighbourhood, expressed in quartiles (Q1 (referent): least deprived, Q2: somewhat deprived, Q3: deprived, Q4: most deprived).

### Covariates

Demographic factors included age, sex (male/female) and ethnic background (white, black and Asian cohorts). Health-related factors included body mass index (BMI; weight in kilograms/height in metres squared) and smoking status (current, past and never).

### Statistical analyses

Descriptive statistics were performed for individual-level characteristics based on the overall sample, mortality, and CVD incidence. Multivariable Cox proportional hazards regression models were used to estimate HRs and 95% CIs in the associations between deprived neighbourhoods and all-cause mortality, incident total CVD, IHD, and CeVD. Furthermore, analyses were performed separately and concurrently to evaluate the differential effect of sex and ethnic background on these associations, adjusting for all covariates. All analyses were conducted using the R software ‘Survival’ package (‘coxph’ and ‘survreg’, version 4.2.2; www.r-project.org). The statistical tests were two sided, and statistical significance was defined as p<0.05. Sensitivity analyses were performed in the overall models adjusted for age, sex and ethnicity, and stratified analyses were adjusted for combinations of age and sex or ethnicity (online supplemental tables 5–8). Additional sensitivity analyses were performed with tertile NDI (least, somewhat and most deprived neighbourhoods) on each outcome (online supplemental tables 9–12).

## Results

### Study participant characteristics

Overall, 261 954 participants were, on average, 55.1 years (SD±8.1), and over half (53.5%) were female, predominantly white cohort (96.2%, table 1). Participants had a mean BMI of 26.8 (SD±4.4), and most were non-smokers (58.0%). Over the follow-up years, 3.8% (n=9933) died and 24.7% (n=64 748) experienced total CVD events, respectively. Those with all-cause mortality and total CVD incidence tended to be older, males, predominantly white participants (96–98%), had higher BMI and were current and former smokers than those without all-cause mortality and incident total CVD. Participants had a mean follow-up of 14.3 years (SD±1.3) with 3 745 307 person-years for all-cause mortality and 12.7 years (SD±3.7) with 3 321 619 person-years for total CVD incidence. All-cause mortality and total CVD incidence rates were 2.65 deaths/1000 person-years and 19.49 events/1000 person-years (table 2).

**Table 1:** Participant characteristics of the UK Biobank cohort, overall and by total cardiovascular disease (CVD) incidence and all-cause mortality status

	Overall, N (%)	All-cause mortality		Total CVD incidence	
	n=261 954	Yes (n=9933; 3.79%)	No (n=252 021; 96.21%)	Yes (n=64 748; 24.72%)	No (n=197 206; 75.28%)
**Demographics**					
Age, M (±SD)	55.13 (8.08)	60.60 (6.79)	54.92 (8.05)	58.51 (7.44)	54.02 (7.98)
Sex					
Male	121 771 (46.49)	5960 (60.0)	115 811 (45.95)	35 490 (54.81)	86 281 (43.75)
Female	140 183 (53.51)	3973 (40.0)	136 210 (54.04)	29 258 (45.19)	110 925 (56.25)
Ethnic composition					
White cohort	252 060 (96.23)	9731 (97.97)	242 329 (96.15)	62 062 (95.85)	189 998 (96.34)
Black cohort	4568 (1.74)	92 (0.93)	4476 (1.78)	1242 (1.92)	3326 (1.69)
Asian cohort	5326 (2.03)	110 (1.10)	5216 (2.07)	1444 (2.23)	3882 (1.97)
**Health-related factors**					
Body mass index, M (±SD)	26.79 (4.37)	27.31 (4.36)	26.77 (4.65)	27.92 (4.64)	26.42 (4.21)
Smoking status					
Former	83 521 (31.88)	3712 (37.37)	70 809 (31.67)	23 653 (36.53)	59 868 (30.36)
Current	26 428 (10.09)	1944 (19.57)	24 484 (9.71)	7376 (11.39)	19 052 (9.66)
Never	152 005 (58.03)	4277 (43.06)	147 728 (58.62)	33 719 (52.08)	118 286 (59.98)

M, mean; SD, standard deviation.

**Table 2:** Longitudinal associations of deprived neighbourhoods with all-cause mortality, incident total cardiovascular disease, ischaemic heart disease and cerebrovascular disease

	All-cause mortality	Total CVD incidence	IHD incidence	CeVD incidence
Events/total participants	9933/261 954	64 748/261 954	15 061/298 992	6729/305 309
Rates†	2.65	19.49	3.55	1.53
**NDI quartile (Q)**	**HR (95% CI)**	**HR (95% CI)**	**HR (95% CI)**	**HR (95% CI)**
Q1 Least deprived	**Referent**			
Q2 Somewhat deprived	1.00 (0.95 to 1.06)	1.02 (1.00 to 1.05)*	1.01 (0.96 to 1.06)	1.09 (1.02 to 1.17)*
Q3 Deprived	1.08 (1.02 to 1.14)**	1.04 (1.02 to 1.07)***	1.03 (0.98 to 1.08)	1.09 (1.02 to 1.17)*
Q4 Most deprived	1.31 (1.24 to 1.38)***	1.13 (1.10 to 1.15)***	1.15 (1.10 to 1.20)***	1.34 (1.25 to 1.44)***
P trend‡	**<0.001**	**<0.001**	**<0.001**	**<0.001**

Models adjusted for age, sex, ethnicity, body mass index (BMI) and smoking.

**Boldface** indicates a significant association.

Significance: *p<0.05; **p<0.01; ***p<0.001.

†Mortality or incidence rates were based on deaths or events/1000 person-years.

‡A linear trend (p trend) was assessed to determine whether there is a significant linear trend in the HRs across NDI in quartiles.

CeVD, cerebrovascular disease; CVD, cardiovascular disease; IHD, ischaemic heart disease; NDI, Neighbourhood Deprivation Index.

The characteristics of participants with and without incident IHD and CeVD were similar to those of all-cause mortality and total CVD incidents (online supplemental table 1). Participants had a mean follow-up of 14.2 years (SD±1.8) with 4 246 447 person-years for IHD and 14.4 years (SD±1.1) with 4 405 552 person-years for CeVD incidence. IHD and CeVD incidence rates were 3.55 and 1.53 events/1000 person-years, respectively (table 2). A similar proportion of participants resided in each quartile of NDI for each outcome (online supplemental tables 2–4).

### Overall associations with all-cause mortality and incident CVD outcomes

Compared with the least deprived, participants residing in the most deprived and deprived neighbourhoods had greater risks of all-cause mortality (HR=1.31, 95% CI 1.24 to 1.38 and HR=1.08, 95% CI 1.02 to 1.14, respectively, table 2). Participants in the most deprived to somewhat deprived neighbourhoods had greater risks of total CVD incidence (HR range: 1.02–1.13). Participants in the most deprived neighbourhoods had a greater risk of IHD incidence (HR=1.15, 95% CI 1.10 to 1.20). Those in the most deprived to somewhat deprived neighbourhoods had greater risks of CeVD incidence (HR range: 1.09–1.34).

### Sex-specific associations with all-cause mortality and incident CVD outcomes

Female participants living in the most deprived compared with the least deprived neighbourhoods had a greater risk of all-cause mortality (HR=1.19, 95% CI 1.09 to 1.31, table 3), more pronounced in males (HR=1.39, 95% CI 1.29 to 1.49). Females in the most deprived and deprived neighbourhoods had greater risks of total CVD incidence (HR range: 1.05–1.13), and similar risks were observed in males (HR range: 1.04–1.13). Females in the deprived neighbourhoods had greater risks of IHD incidence (HR range: 1.09–1.27), less pronounced in males (HR=1.09). Females in the most deprived and deprived neighbourhoods had greater risks of CeVD incidence (HR range: 1.14–1.34). Males in the most deprived and somewhat deprived neighbourhoods had greater risks of CeVD incidence (HR range: 1.10–1.34).

**Table 3:** Sex-specific longitudinal associations of deprived neighbourhoods with all-cause mortality, incident total cardiovascular disease, ischaemic heart disease and cerebrovascular disease

	All-cause mortality	
	Female	Male
Events/total participants	3973/140 183	5960/121 771
Rates†	1.97	3.44
**NDI quartile (Q)**	**HR (95% CI)**	**HR (95% CI)**
Q1 Least deprived	**Referent**	
Q2 Somewhat deprived	0.97 (0.89 to 1.06)	1.02 (0.95 to 1.10)
Q3 Deprived	1.04 (0.95 to 1.14)	1.10 (1.03 to 1.19)**
Q4 Most deprived	1.19 (1.09 to 1.31)***	1.39 (1.29 to 1.49)***
P trend‡	**<0.001**	**<0.001**
	**Total cardiovascular disease incidence**	
Events/total participants	29 258/140 183	35 490/121 771
Rates†	16.06	23.66
Q1 Least deprived	**Referent**	
Q2 Somewhat deprived	1.02 (0.99 to 1.05)	1.03 (1.00 to 1.06)^+^
Q3 Deprived	1.05 (1.01 to 1.08)**	1.04 (1.01 to 1.07)**
Q4 Most deprived	1.13 (1.09 to 1.17)***	1.13 (1.09 to 1.16)***
P trend‡	**<0.001**	**<0.001**
	**Ischaemic heart disease incidence**	
Events/total participants	4914/158 879	10 147/140 113
Rates†	2.16	5.16
Q1 Least deprived	**Referent**	
Q2 Somewhat deprived	1.08 (0.99 to 1.17)^+^	0.98 (0.93 to 1.04)
Q3 Deprived	1.09 (1.01 to 1.19)*	1.00 (0.95 to 1.06)
Q4 Most deprived	1.27 (1.17 to 1.38)***	1.09 (1.03 to 1.15)**
P trend‡	**<0.001**	**0.002**
	**Cerebrovascular disease incidence**	
Events/total participants	2648/160 649	4081/144 660
Rates†	1.14	1.96
Q1 Least deprived	**Referent**	
Q2 Somewhat deprived	1.08 (0.97 to 1.21)	1.10 (1.00 to 1.20)*
Q3 Deprived	1.14 (1.02 to 1.27)*	1.06 (0.97 to 1.16)
Q4 Most deprived	1.34 (1.20 to 1.49)***	1.34 (1.23 to 1.46)***
P trend‡	**<0.001**	**<0.001**

Models adjusted for age, ethnicity, body mass index (BMI) and smoking.

**Boldface** indicates a significant association.

Significance: *p<0.05; **p<0.01; ***p<0.001 and +p<0.1.

†Mortality or incidence rates were based on deaths or events/1000 person-years.

‡A linear trend (p trend) was assessed to determine whether there is a significant linear trend in the HRs across NDI in quartiles.

NDI, Neighbourhood Deprivation Index.

### Ethnicity-specific associations with all-cause mortality and incident CVD outcomes

The white cohort living in the most deprived and deprived neighbourhoods compared with the least deprived had greater risks of all-cause mortality (HR=1.31, 95% CI 1.24 to 1.39 and HR=1.08, 95% CI 1.02 to 1.14, respectively, table 4). Similarly, the white cohort in the most deprived to somewhat deprived neighbourhoods had greater risks of total CVD incidence (HR range: 1.02–1.13). The white participants in the most deprived neighbourhoods had a greater risk of IHD incidence (HR=1.15). The white cohort in the deprived neighbourhoods had great risks of CeVD incidence (HR range: 1.09–1.34). NDI was not associated with each outcome among the black and Asian cohorts.

**Table 4:** Ethnicity-specific longitudinal associations of deprived neighbourhoods with all-cause mortality, incident total cardiovascular disease, ischaemic heart disease and cerebrovascular disease

	All-cause mortality		
	White Cohort	Black Cohort	Asian Cohort
Events/total participants	9731/252 060	92/4568	110/5326
Rates†	2.70	1.40	1.43
**NDI quartile (Q)**	**HR (95% CI)**	**HR (95% CI)**	**HR (95% CI)**
Q1 Least deprived	**Referent**		
Q2 Somewhat deprived	1.00 (0.95 to 1.06)	1.17 (0.29 to 4.69)	0.86 (0.43 to 1.70)
Q3 Deprived	1.08 (1.02 to 1.14)**	1.14 (0.33 to 3.93)	0.63 (0.33 to 1.19)
Q4 Most deprived	1.31 (1.24 to 1.39)***	1.16 (0.36 to 3.70)	0.87 (0.50 to 1.53)
P trend‡	**<0.001**	0.727	0.777
	**Total cardiovascular disease incidence**		
Events/total participants	62 062/252 060	1242/4568	1444/5326
Rates†	19.40	21.84	21.81
Q1 Least deprived	**Referent**		
Q2 Somewhat deprived	1.02 (1.00 to 1.05)*	0.99 (0.68 to 1.42)	1.04 (0.85 to 1.29)
Q3 Deprived	1.04 (1.02 to 1.07)***	0.91 (0.65 to 1.25)	1.07 (0.89 to 1.29)
Q4 Most deprived	1.13 (1.10 to 1.15)***	1.08 (0.80 to 1.45)	1.13 (0.95 to 1.35)
P trend‡	**<0.001**	0.138	0.110
	**Ischaemic heart disease incidence**		
Events/total participants	14 390/287 358	170/5441	501/6193
Rates†	3.53	2.18	5.80
Q1 Least deprived	**Referent**		
Q2 Somewhat deprived	1.01 (0.97 to 1.06)	1.02 (0.34 to 3.05)	0.81 (0.57 to 1.15)
Q3 Deprived	1.04 (0.99 to 1.09)	0.69 (0.25 to 1.89)	0.84 (0.62 to 1.14)
Q4 Most deprived	1.15 (1.10 to 1.20)***	1.38 (0.57 to 3.38)	0.91 (0.69 to 1.21)
P trend‡	**<0.001**	0.053	0.984
	**Cerebrovascular disease incidence**		
Events/total participants	6449/293 301	143/5511	137/6497
Rates†	1.52	1.80	1.46
Q1 Least deprived	**Referent**		
Q2 Somewhat deprived	1.09 (1.02 to 1.17)*	0.69 (0.21 to 2.26)	1.12 (0.52 to 2.41)
Q3 Deprived	1.09 (1.02 to 1.17)*	1.13 (0.43 to 2.95)	1.13 (0.57 to 2.26)
Q4 Most deprived	1.34 (1.25 to 1.43)***	1.10 (0.45 to 2.70)	1.69 (0.90 to 3.18)
P trend‡	**<0.001**	0.485	**0.028**

Models adjusted for age, sex, body mass index (BMI) and smoking.

**Boldface** indicates a significant association.

Significance: *p<0.05; **p<0.01; and ***p<0.001.

†Mortality or incidence rates were based on deaths or events/1000 person-years.

‡A linear trend (p trend) was assessed to determine whether there is a significant linear trend in the HRs across NDI in quartiles.

NDI, Neighbourhood Deprivation Index.

### Sex- and ethnicity-specific associations with all-cause mortality and incident CVD outcomes

The white female participants residing in the deprived neighbourhoods had a greater risk of all-cause mortality (HR=1.20) and more pronounced in males (HR=1.39, table 5). The white females and males in the deprived neighbourhoods had greater risks of total CVD incidence (female HR range: 1.04–1.13 and male HR range: 1.04–1.13). The white females in the deprived neighbourhoods had greater risks of IHD incidence (HR range: 1.09–1.27) and less pronounced in males (HR=1.09). Furthermore, the white females in the most deprived and deprived neighbourhoods had greater risks of CeVD incidence (HR range: 1.14–1.33). In turn, the white males in the most deprived and somewhat deprived neighbourhoods had greater risks of CeVD incidence (HR range: 1.10–1.33). Overall, the black and Asian females and males in the deprived neighbourhoods did not have a greater risk for each outcome with the exception of Asian females in the most deprived neighbourhoods (HR=1.37).

**Table 5:** Sex and ethnicity-specific longitudinal associations of deprived neighbourhoods with all-cause mortality, incident total cardiovascular disease, ischaemic heart disease and cerebrovascular disease

	All-cause mortality					
	White cohort		Black cohort		Asian cohort	
	Female	Male	Female	Male	Female	Male
Events/total participants	3894/135 219	5837/116 841	38/2468	54/2100	41/2496	69/2830
Rates†	2.01	3.51	1.07	1.79	1.13	1.70
**NDI quartile (Q)**	**HR (95% CI)**	**HR (95% CI)**	**HR (95% CI)**	**HR (95% CI)**	**HR (95% CI)**	**HR (95% CI)**
Q1 Least deprived	**Referent**					
Q2 Somewhat deprived	0.97 (0.88 to 1.06)	1.02 (0.95 to 1.10)	1.12 (0.22 to 6.57)	1.12 (0.10 to 12.40)	1.21 (0.38 to 3.84)	0.71 (0.30 to 1.67)
Q3 Deprived	1.04 (0.95 to 1.14)	1.11 (1.03 to 1.19)**	0.55 (0.11 to 2.85)	2.25 (0.29 to 17.59)	0.75 (0.25 to 2.23)	0.57 (0.26 to 1.26)
Q4 Most deprived	1.20 (1.10 to 1.31)***	1.39 (1.30 to 1.50)***	0.67 (0.16 to 2.85)	2.05 (0.28 to 14.94)	1.10 (0.41 to 2.93)	0.78 (0.39 to 1.54)
P trend‡	**<0.001**	**<0.001**	0.474	0.322	0.910	0.671
	**Total cardiovascular heart disease incidence**					
Events/total participants	28 005/135 219	34 057/116 841	667/2468	575/2100	586/2496	858/2830
Rates†	15.92	23.66	21.66	22.06	18.45	24.92
Q1 Least deprived	**Referent**					
Q2 Somewhat deprived	1.02 (0.98 to 1.05)	1.03 (1.00 to 1.06)^+^	0.80 (0.49 to 1.30)	1.26 (0.72 to 2.19)	1.26 (0.90 to 1.77)	0.93 (0.71 to 1.21)
Q3 Deprived	1.04 (1.01 to 1.08)**	1.04 (1.01 to 1.07)**	0.85 (0.56 to 1.30)	0.99 (0.60 to 1.65)	1.35 (1.00 to 1.82)^+^	0.92 (0.72 to 1.17)
Q4 Most deprived	1.13 (1.09 to 1.16)***	1.13 (1.09 to 1.16)***	0.95 (0.65 to 1.41)	1.27 (0.80 to 2.04)	1.37 (1.03 to 1.83)*	1.00 (0.80 to 1.24)
P trend‡	**<0.001**	**<0.001**	0.422	0.152	**0.043**	0.717
	**Ischaemic heart disease incidence**					
Events/total participants	4685/153 046	9705/134 312	82/2955	88/2486	147/2878	354/3315
Rates†	2.13	5.15	1.93	2.47	3.60	7.77
Q1 Least deprived	**Referent**					
Q2 Somewhat deprived	1.09 (1.00 to 1.18)*	0.98 (0.93 to 1.04)	0.37 (0.06 to 2.20)	2.03 (0.42 to 9.78)	0.59 (0.29 to 1.21)	0.90 (0.60 to 1.34)
Q3 Deprived	1.10 (1.02 to 1.20)*	1.01 (0.95 to 1.06)	0.66 (0.18 to 2.43)	0.69 (0.14 to 3.41)	0.87 (0.49 to 1.52)	0.83 (0.58 to 1.20)
Q4 Most deprived	1.27 (1.16 to 1.38)***	1.09 (1.03 to 1.16)**	1.04 (0.32 to 3.33)	1.91 (0.47 to 7.79)	1.12 (0.67 to 1.87)	0.84 (0.60 to 1.17)
P trend‡	**<0.001**	**0.001**	0.161	0.166	0.168	0.331
	**Cerebrovascular disease incidence**					
Events/total participants	2553/154 702	3896/138 599	61/2996	82/2515	34/2951	103/3546
Rates†	1.14	1.95	1.41	2.28	0.79	2.02
Q1 Least deprived	**Referent**					
Q2 Somewhat deprived	1.08 (0.97 to 1.21)	1.10 (1.01 to 1.20)*	0.51 (0.10 to 2.56)	0.93 (0.16 to 5.57)	2.17 (0.42 to 11.17)	0.90 (0.37 to 2.17)
Q3 Deprived	1.14 (1.02 to 1.28)*	1.06 (0.96 to 1.16)	0.68 (0.18 to 2.48)	1.82 (0.42 to 7.96)	1.51 (0.31 to 7.29)	1.05 (0.49 to 2.26)
Q4 Most deprived	1.33 (1.19 to 1.49)***	1.33 (1.22 to 1.46)***	0.68 (0.21 to 2.21)	1.69 (0.41 to 6.96)	2.74 (0.64 to 11.78)	1.46 (0.72 to 2.95)
P trend‡	**<0.001**	**<0.001**	0.875	0.334	0.149	0.094

Models adjusted for age, body mass index (BMI) and smoking.

**Boldface** indicates a significant association.

Significance: *p<0.05; **p<0.01; ***p<0.001 and +p<0.1.

†Mortality or incidence rates were based on deaths or events/1000 person-years.

‡A linear trend (p trend) was assessed to determine whether there is a significant linear trend in the HRs across NDI in quartiles.

NDI, Neighbourhood Deprivation Index.

### Sensitivity analyses

Sensitivity analyses were performed for each analysis, and the effect sizes of the associations were stronger than those controlled for all covariates (online supplemental tables 5–8). Overall, the risk of each outcome based on tertile NDI was consistent with the results of quartile NDI (online supplemental tables 9–12).

## Discussion

This study demonstrated that living in deprived neighbourhoods had a greater risk of all-cause mortality and incident total CVD, IHD and CeVD using over 250 000 UK Biobank participants. Similar adverse associations for each outcome were found for both sexes, yet the strength of the associations varied by each outcome. Furthermore, only the white participants in the deprived neighbourhoods had a greater risk of each outcome, but not for the black and Asian participants. The white females and males in the most deprived neighbourhoods had a greater risk of each outcome. However, such associations were not observed for the black and Asian cohorts, except for total CVD incidence among Asian females.

### All-cause mortality stratified by sex and ethnic background separately and concurrently

Consistent with the previous research,^16 17^ this study indicated that those in the most deprived neighbourhoods had a greater risk of all-cause mortality. For example, previous studies showed that residing in the most deprived neighbourhoods^16^ or low individual-level SES adjusting for NDI^17^ was associated with a greater risk of all-cause mortality. A possible explanation may be that this study and the other two studies used UK Biobank data and assessed NDI similarly, with consistent inclusion and exclusion criteria. Alternatively, individuals with high SES tended to have better health, leading to longevity, which, in turn, could afford to live in advantaged neighbourhoods with better access to healthcare resources and promote healthy lifestyles.^17^

Furthermore, these associations varied by sex. This study indicated that living in the most deprived neighbourhoods had a greater risk of all-cause mortality among males than females (HR: 1.39 vs 1.19). Similarly, one US study indicated that males in the most deprived neighbourhoods had a higher risk of all-cause mortality than females.^18^ Yet, the other US study showed that females in the most deprived neighbourhoods had a greater risk of mortality but not males.^19^ It may be due to differences by sex in responses to neighbourhood environments (access to greenspace) and coping mechanisms for environmental stressors (crime, poverty).^19^ Furthermore, previous UK studies on health behaviours and mortality indicated that males had a greater risk of mortality due to smoking and more alcohol consumption^20^ than females. These behavioural differences by sex may be an alternative reason why males had a greater risk of all-cause mortality than females.

Inconsistent with the previous studies,^21 22^ the present study indicated that only the white cohort in the most deprived neighbourhoods had a greater risk of all-cause mortality, but not for the black and Asian cohorts. For instance, one US study found that compared with white counterparts living in the most deprived neighbourhoods, minority groups (eg, black adults) had a greater risk of mortality.^21^ A possible explanation could be that the UK and US populations may vary by racial and ethnic compositions, and their demographic characteristics (education) and social norms about health (smoking).^23^ Ethnicity-stratified analyses using the UK Biobank remained scarce. Future research should consider the role of ethnic background.

This study may be one of the first studies using the UK Biobank data to address disparities by sex and ethnic background simultaneously. Inconsistent with other studies,^19^ only white females and males in the most deprived neighbourhoods had a greater risk of all-cause mortality, but not other cohorts. One US study indicated that white and black males and females in the most deprived neighbourhoods were not related to all-cause mortality, except for black females.^19^ The reason for this discrepancy could be the different societal structure (UK vs USA) and the fewer cases of all-cause mortality in the black and Asian males and females compared with their white counterparts (table 1). Future studies should consider oversampling black and Asian groups from the least and somewhat deprived neighbourhoods (online supplemental tables 2–4).

### Incident CVD outcomes stratified by sex and ethnic background separately and concurrently

Comparable to the previous UK Biobank studies,^16 17^ individuals in deprived neighbourhoods had greater risks for each CVD outcome. For instance, previous UK studies indicated that individuals with low SES (adjusting for neighbourhood SES) and living in highly deprived neighbourhoods had a greater risk of overall CVD incidence^17^ and coronary artery disease.^16^ A possible explanation was that these studies used similar definitions of CVD outcomes with similar lengths of follow-up years.^16 17^

In sex-specific analyses, this study showed that females and males living in the most deprived neighbourhoods were associated with a greater risk of each CVD outcome. The strength of the associations was larger among females than males for IHD (HR: 1.27 vs 1.09). This result was somewhat consistent with the US study indicating that living in deprived neighbourhoods for over 10 years was related to a higher risk of CVD mortality, more pronounced in females than males.^10^ Furthermore, Swedish studies showed that females in deprived neighbourhoods had an increased risk for incidentcoronary heart disease than males.^24 25^ Additionally, females and males in deprived neighbourhoods had a higher risk for CeVD, inconsistent with previous research.^26^,^28^ A review reported mixed results on neighbourhood deprivation and stroke.^26^ One Swedish study found that living in disadvantaged neighbourhoods increased the risk of ischaemic stroke incidence for both sexes.^27^ However, one US study indicated the null findings.^28^ Our results might be more consistent with those of the Swedish study^27^ due to the analysis controlling for similar characteristics and participants from Europe versus USA.^26^

In ethnicity-specific analysis, the white cohort living in the most deprived neighbourhoods had a greater risk of each CVD outcome, yet not for the black and Asian cohorts, inconsistent with the previous UK^29^ and US^30 31^ studies. For example, one US study showed that in the fully adjusted models, low neighbourhood SES was not related to incident stroke among white and black individuals.^30^ Taken together, the inconsistencies among racial and ethnic groups may be due to differences in study sites (UK^29^ vs across US states^30 31^), various age groups (middle to older^29 30^ vs older^31^) and covariates (age, sex,^29^ region,^30^ SES^30^ and behavioural and biological factors^31^). Additionally, fewer black and Asian participants in less deprived areas could contribute to null findings (online supplemental tables 2–4). From a public health perspective, the adverse effects of deprived neighbourhoods on CVD may begin in early adulthood; therefore, efforts to invest in low SES areas should be emphasised to reduce the burden of CVD,^10^ particularly for minority individuals from a wide range of age groups.

The present study may be the first to investigate sex and ethnicity-stratified associations using UK Biobank data. Only white females and males and Asian females in the most deprived neighbourhoods had a greater risk of total CVD incidence, consistent with previous studies.^32 33^ The present study showed that deprived neighbourhoods were not related to IHD incidence among black males and females. One study showed that lower neighbourhood SES was related to stroke incidence only among black females but not for black males, and white males and females.^32^ In sum, sex and ethnicity-stratified analyses revealed inconsistencies with previous studies, and these analyses have been highly understudied. Further studies on this line of research would be warranted to better elucidate how living in deprived neighbourhoods may impact CVD outcomes by racial and ethnic groups.

### Strengths and limitations

The major strength of this study included the large prospective cohort design, providing sufficient cases to investigate the longitudinal associations for each outcome and adjusting for various covariates. Several limitations need to be addressed. First, the UK Biobank participants were predominantly white ethnic background, health-conscious individuals in wealthier neighbourhoods, which could lead to an underestimation of the association. This may not be applicable to the general UK population.^16^ Second, this study was observational; therefore, the results do not infer causality. Third, other residual and unmeasured confounders might not be controlled in the model (ie, familial predisposition^34^), which could be related to the outcomes. To minimise this issue, we performed a sensitivity analysis of how removing health-related factors (BMI, smoking) may impact the estimates for the analyses. Fourth, NDI was an area-level measure based on the postal region, which differed from individual-level SES. Thus, interpreting the results should be cautious as this study did not control individual SES. Future studies should also consider various social deprivation indices with all-cause mortality and CVD outcomes. Fifth, the UK Biobank does not represent the general UK population, which tends to be older, include more females and live in less deprived neighbourhoods compared with those who did not participate, resulting in selection bias towards healthy volunteers.^35^ Lastly, due to fewer participants in less deprived areas among black and Asian cohorts, further research should consider sampling frames for different deprivation areas.

## Conclusions

This study demonstrated that living in deprived neighbourhoods was associated with a higher risk of each outcome. Similar patterns of the associations were found by sex in the UK Biobank participants. In particular, the white male and female cohort living in the deprived neighbourhoods had a greater risk of these outcomes, but not for the black and Asian cohorts, except for Asian female adults. These findings may shed light on how sex and ethnic background could play an important role in elucidating the association between deprived neighbourhoods and chronic diseases at the population level.

## Supplementary material

10.1136/openhrt-2025-003225 online supplemental file 1

## Data Availability

Data may be obtained from a third party and are not publicly available.
