# Supplementary material for: Neighbourhood deprivation and cardiometabolic outcomes in the UK Biobank: differences by sex and ethnicity
Source: Open Heart. 2025 May 27;12(1):e003225. doi: 10.1136/openhrt-2025-003225 (PMC12121578; doi:10.1136/openhrt-2025-003225)
Supplement: online supplemental file 1 [file openhrt-12-1-s001.docx]

| **Supplemental Table 1.** Baseline participants' characteristics of overall, ischemic heart disease, and cerebrovascular disease by incidence status | | | | | | |
| --- | --- | --- | --- | --- | --- | --- |
|  | Overall, n (%) | IHD incidence, n (%) | | Overall, n (%) | CeVD Incidence, n (%) | |
|  | N=298,992 | Yes (n=15,061; 5.04%) | No (n=283,931; 94.96%) | N=305,309 | Yes (n=6,729; 2.20%) | No (n=298,580; 97.80%) |
| **Demographics** |  |  |  |  |  |  |
| Age, M (±SD) | 55.54 (8.09) | 59.61 (7.06) | 55.33 (8.09) | 55.67 (8.10) | 61.10 (6.73) | 55.55 (8.09) |
| Sex |  |  |  |  |  |  |
| Male | 140,113 (46.86) | 10,147 (67.37) | 129,966 (45.77) | 144,660 (47.38) | 4,081 (60.65) | 140,579 (47.08) |
| Female | 158,879 (53.14) | 4,914 (32.63) | 153,965 (54.23) | 160,649 (52.62) | 2,648 (39.35) | 158,001 (52.92) |
| Race/ethnicity |  |  |  |  |  |  |
| White cohort | 287,358 (96.11) | 14,390 (95.54) | 272,968 (96.14) | 293,301 (96.07) | 6,449 (95.84) | 286,852 (96.07) |
| Black cohort | 5,441(1.82) | 170 (1.13) | 5,271 (1.86) | 5,511 (1.81) | 143 (2.13) | 5,368 (1.80) |
| Asian cohort | 6,193 (2.07) | 501 (3.33) | 5,692 (2.00) | 6,497 (2.13) | 137 (2.04) | 6,360 (2.13) |
| **Health-related factors** |  |  |  |  |  |  |
| Body mass index, M (±SD) | 26.96(4.47) | 28.22 (4.57) | 26.89 (4.46) | 27.00 (4.48) | 27.64 (4.67) | 26.98 (4.48) |
| Smoking status |  |  |  |  |  |  |
| Former | 97,467 (32.60) | 5,824 (38.67) | 91,643 (32.28) | 100,409 (32.89) | 2,550 (37.90) | 97,859 (32.77) |
| Current | 30,065 (10.06) | 2,102 (13.96) | 27,963 (9.85) | 30,659 (10.04) | 1035 (15.38) | 29,624 (9.92) |
| Never | 171,460 (57.35) | 7,135 (47.37) | 164,325 (57.87) | 174,241 (57.07) | 3,144 (46.72) | 171,097 (57.30) |
| **Note**: Abbreviations: M (±SD), Mean and standard deviation; IHD, Ischemic heart disease; CeVD, Cerebrovascular disease | | | | | | |

| **Supplemental Table 2.** Participants' characteristics for all-cause mortality and incident total cardiovascular disease, by neighborhood deprivation index (n=261,954) | | | | |
| --- | --- | --- | --- | --- |
|  | Q1 (Least deprived) n=66,320 (25.32%) | Q2 (Somewhat deprived) n=65,792 (25.12%) | Q3 (Deprived) n=65,531 (25.02%) | Q4 (Most deprived) n=64,311 (24.55%) |
| **Demographics** |  |  |  |  |
| Age, M (±SD) | 55.91 (7.89) | 55.72 (7.98) | 54.99 (8.10) | 53.87 (8.21) |
| Sex |  |  |  |  |
| Male | 30,882 (46.57) | 30,215 (45.93) | 29,976 (45.74) | 30,698 (47.73) |
| Female | 35,438 (53.43) | 35,577 (54.07) | 35,555 (54.26) | 33,613 (52.27) |
| Race/ethnicity |  |  |  |  |
| White cohort | 65,555 (98.85) | 64,741 (98.40) | 63,327 (96.64) | 58,437 (90.87) |
| Black cohort | 175 (0.26) | 290 (0.44) | 788 (1.20) | 3,315 (5.15) |
| Asian cohort | 590 (0.89) | 761 (1.16) | 1,416 (2.16) | 2,559 (3.98) |
| **Health-related factors** |  |  |  |  |
| Body mass index, M (±SD) | 26.50 (4.07) | 26.68 (4.20) | 26.82 (4.37) | 27.19 (4.80) |
| Smoking status |  |  |  |  |
| Former | 20,456 (30.84) | 20,903 (31.77) | 21,349 (32.58) | 20,813 (32.36) |
| Current | 4,261 (6.42) | 4,973 (7.56) | 6,537(9.98) | 10,657 (16.57) |
| Never | 41,603 (62.73) | 39,916 (60.67) | 37,645 (57.45) | 32,841 (51.07) |
| **Note**: Abbreviations: M (±SD), Mean and standard deviation; Q: quartile. | | | | |

| **Supplemental Table 3.** Participants' characteristics for incident ischemic heart disease by neighborhood deprivation (n=298,992) | | | | |
| --- | --- | --- | --- | --- |
|  | Q1 (Least deprived) n=74,990 (25.08%) | Q2 (Somewhat deprived) n=75,064 (25.11%) | Q3 (Deprived) n=74,770 (25.00%) | Q4 (Most deprived) n=74,168 (24.81%) |
| **Demographics** |  |  |  |  |
| Age, M (±SD) | 56.27 (7.87) | 56.12 (7.97) | 55.42 (8.11) | 54.34 (8.27) |
| Sex |  |  |  |  |
| Male | 35,172 (46.90) | 34,763 (46.31) | 34,438 (46.06) | 35,740 (48.19) |
| Female | 39,818 (53.09) | 40,301 (53.69) | 40,332 (53.94) | 38,428 (51.81) |
| Race/ethnicity |  |  |  |  |
| White cohort | 74,117 (98.84) | 73,829 (98.35) | 72,188 (96.55) | 67,224 (90.64) |
| Black cohort | 205 (0.27) | 351 (0.47) | 935 (1.25) | 3,950 (5.33) |
| Asian cohort | 668 (0.89) | 884 (1.18) | 1,647 (2.20) | 2,994 (4.04) |
| **Health-related factors** |  |  |  |  |
| Body mass index, M (±SD) | 26.62 (4.14) | 26.82 (4.28) | 26.99 (4.47) | 27.41 (4.92) |
| Smoking status |  |  |  |  |
| Former | 23,646 (31.53) | 24,356 (32.45) | 24,978 (33.41) | 24,487 (33.01) |
| Current | 4,823 (6.43) | 5,669 (7.55) | 7,373 (9.86) | 12,200 (16.45) |
| Never | 46,521 (62.04) | 45,039 (60.00) | 42,419 (56.73) | 37,481 (50.54) |
| **Note**: Abbreviations: M (±SD), Mean and standard deviation; Q: quartile. | | | | |

| **Supplemental Table 4.** Participants' characteristics for incident cerebrovascular disease, by neighborhood deprivation (n=305,309) | | | | |
| --- | --- | --- | --- | --- |
|  | Q1 (Least deprived) n=76,548 (25.07%) | Q2 (Somewhat deprived) n=76,486 (25.05%) | Q3 (Deprived) n=76,348 (25.01%) | Q4 (Most deprived) n=75,927 (24.87%) |
| **Demographics** |  |  |  |  |
| Age, M (±SD) | 56.38 (7.87) | 56.24 (7.97) | 55.55 (8.13) | 54.50 (8.29) |
| Sex |  |  |  |  |
| Male | 36,294 (47.41) | 35,834 (46.85) | 35,564 (46.58) | 36,968 (48.69) |
| Female | 40,254 (52.59) | 40,652 (53.15) | 40,784 (53.42) | 38,959 (51.31) |
| Race/ethnicity |  |  |  |  |
| White cohort | 75,643 (98.82) | 75,212 (98.33) | 73,664 (96.48) | 68,782 (90.59) |
| Black cohort | 207 (0.27) | 354 (0.46) | 951 (1.25) | 3,999 (5.27) |
| Asian cohort | 698 (0.91) | 920 (1.20) | 1,733 (2.27) | 3,146 (4.14) |
| **Health-related factors** |  |  |  |  |
| Body mass index, M (±SD) | 26.65 (4.14) | 26.85 (4.29) | 27.03 (4.48) | 27.45 (4.93) |
| Smoking status |  |  |  |  |
| Former | 24,363 (31.83) | 25,075 (32.78) | 25,718 (33.69) | 25,253 (33.26) |
| Current | 4,912 (6.42) | 5,751 (7.52) | 7,535 (9.87) | 12,461 (16.41) |
| Never | 47,273 (61.76) | 45,660 (59.70) | 43,095 (56.45) | 38,213 (50.33) |
| **Note**: Abbreviations: M (±SD), Mean and standard deviation; Q: quartile. | | | | |

| **Supplemental Table 5.** Longitudinal associations of deprived neighborhoods with all-cause mortality, incident total cardiovascular disease, ischemic heart disease, and cerebrovascular disease | | | | |
| --- | --- | --- | --- | --- |
|  | **All-cause mortality** | **Total CVD incidence** | **IHD incidence** | **CeVD incidence** |
| Events/total participants | 9,933/26,1954 | 64,748/261,954 | 15,061/298,992 | 6,729/305,309 |
| NDI Quartile (Q) | HR (95% CI) | HR (95% CI) | HR (95% CI) | HR (95% CI) |
| Q1 Least deprived | **Referent** | | | |
| Q2 Somewhat deprived | 1.02 (0.96, 1.08) | **1.04 (1.02, 1.06)***** | 1.03 (0.98, 1.08) | **1.11 (1.03, 1.19)**** |
| Q3 Deprived | **1.13 (1.07, 1.20)***** | **1.08 (1.06, 1.10)***** | **1.07 (1.02, 1.12)**** | **1.13 (1.05, 1.21)***** |
| Q4 Most deprived | **1.49 (1.41, 1.58)***** | **1.21 (1.18, 1.24)***** | **1.26 (1.20, 1.32)***** | **1.46 (1.36, 1.56)***** |
| **Note**: Abbreviations: CVD, cardiovascular disease; IHD, ischemic heart disease; CeVD, cerebrovascular disease; Hazard ratio, HR; CI, Confidence interval. Models adjusted for age, sex, and ethnicity. Significance: ***p<.001; **p<.01; *p<.05; and ^+^p<.1. **Boldface** indicates a significant association. | | | | |

| **Supplemental Table 6**. Sex-specific longitudinal associations of deprived neighborhoods with all-cause mortality, incident total cardiovascular disease, ischemic heart disease, and cerebrovascular disease | | |
| --- | --- | --- |
|  | **All-cause mortality** | |
|  | **Female** | **Male** |
| Events/total participants | 3,973/140,183 | 5,960/121,771 |
| NDI Quartile (Q) | HR (95% CI) | HR (95% CI) |
| Q1 Least deprived | **Referent** | |
| Q2 Somewhat deprived | 0.99 (0.91, 1.08) | 1.04 (0.97, 1.22) |
| Q3 Deprived | **1.10 (1.01, 1.20)*** | **1.16 (1.08, 1.25)***** |
| Q4 Most deprived | **1.37 (1.26, 1.50)***** | **1.58 (1.47, 1.69)***** |
|  | **Total cardiovascular disease incidence** | |
| Events/total participants | 29,258/140,183 | 35,490/121,771 |
| Q1 Least deprived | **Referent** | |
| Q2 Somewhat deprived | **1.04 (1.01, 1.07)*** | **1.04 (1.01, 1.07)*** |
| Q3 Deprived | **1.09 (1.06, 1.13)***** | **1.07 (1.04, 1.10)***** |
| Q4 Most deprived | **1.23 (1.19, 1.27)***** | **1.19 (1.16, 1.23)***** |
|  | **Ischemic heart disease incidence** | |
| Events/total participants | 4,914/158,879 | 10,147/140,113 |
| Q1 Least deprived | **Referent** | |
| Q2 Somewhat deprived | **1.10 (1.02, 1.20)*** | 0.99 (0.94, 1.05) |
| Q3 Deprived | **1.16 (1.07, 1.26)***** | 1.03 (0.98, 1.09) |
| Q4 Most deprived | **1.44 (1.33, 1.56)***** | **1.18 (1.11, 1.24)***** |
|  | **Cerebrovascular disease incidence** | |
| Events/total participants | 2,648/160,649 | 4,081/144,660 |
| Q1 Least deprived | **Referent** | |
| Q2 Somewhat deprived | 1.10 (0.98, 1.23)^+^ | **1.11 (1.02, 1.21)*** |
| Q3 Deprived | **1.19 (1.06, 1.33)**** | 1.09 (1.00, 1.20)^+^ |
| Q4 Most deprived | **1.47 (1.32, 1.64)***** | **1.45 (1.33, 1.58)***** |
| **Note**: Abbreviations: Hazard ratio, HR; CI, Confidence interval. Models adjusted for age and ethnicity. Significance: ***p<.001; **p<.01; *p<.05; and ^+^p<.1. **Boldface** indicates a significant association. | | |

| **Supplemental Table 7.** Ethnic-specific longitudinal associations of deprived neighborhoods with all-cause mortality, incident total cardiovascular disease, ischemic heart disease, and cerebrovascular disease | | | |
| --- | --- | --- | --- |
|  | **All-cause mortality** | | |
|  | **White** | **Black** | **Asian** |
| Events/total participants | 9,731/252,060 | 92/4,568 | 110/5,326 |
| NDI Quartile (Q) | HR (95% CI) | HR (95% CI) | HR (95% CI) |
| Q1 Least deprived | **Referent** | | |
| Q2 Somewhat deprived | 1.02 (0.96, 1.08) | 1.20 (0.30, 4.80) | 0.87 (0.44, 1.72) |
| Q3 Deprived | **1.14 (1.08, 1.21)***** | 1.18 (0.34, 4.08) | 0.64 (0.34, 1.21) |
| Q4 Most deprived | **1.50 (1.42, 1.59)***** | 1.27 (0.40, 4.04) | 0.92 (0.53, 1.62) |
|  | **Total cardiovascular disease incidence** | | |
| Events/total participants | 62,062/252,060 | 1,242/4,568 | 1,444/5,326 |
| Q1 Least deprived | **Referent** | | |
| Q2 Somewhat deprived | **1.04 (1.02, 1.06)***** | 1.03 (0.72, 1.49) | 1.04 (0.84, 1.28) |
| Q3 Deprived | **1.08 (1.06, 1.10)***** | 0.95 (0.69, 1.32) | 1.10 (0.91, 1.32) |
| Q4 Most deprived | **1.21 (1.18, 1.24)***** | 1.16 (0.86, 1.57) | 1.19 (1.00, 1.42)^+^ |
|  | **Ischemic heart incidence** | | |
| Events/total participants | 14,390/287,358 | 170/5,441 | 501/6,193 |
| Q1 Least deprived | **Referent** | | |
| Q2 Somewhat deprived | 1.03 (0.99, 1.08) | 1.05 (0.35, 3.15) | 0.82 (0.58, 1.16) |
| Q3 Deprived | **1.08 (1.03, 1.13)**** | 0.74 (0.27, 2.02) | 0.86 (0.63, 1.16) |
| Q4 Most deprived | **1.26 (1.21, 1.32)***** | 1.56 (0.64, 3.82) | 0.96 (0.72, 1.27) |
|  | **Cerebrovascular disease incidence** | | |
| Events/total participants | 6,449/293,301 | 143/5,511 | 137/6,497 |
| Q1 Least deprived | **Referent** | | |
| Q2 Somewhat deprived | **1.11 (1.03, 1.19)**** | 0.72 (0.22, 2.35) | 1.12 (0.52, 2.41) |
| Q3 Deprived | **1.13 (1.05, 1.21)***** | 1.19 (0.46, 3.11) | 1.16 (0.58, 2.31) |
| Q4 Most deprived | **1.46 (1.36, 1.56)***** | 1.23 (0.50, 3.01) | 1.78 (0.94, 3.34)^+^ |
| **Note**: Abbreviations: Hazard ratio: HR. CI: Confidence interval. Models adjusted for age and sex. Significance: ***p<.001; **p<.01; *p<.05; and ^+^p<.1. **Boldface** indicates a significant association. | | | |

| **Supplemental Table 8**. Sex- and ethnic-specific longitudinal associations of deprived neighborhoods with all-cause mortality, incident total cardiovascular disease, ischemic heart disease, and cerebrovascular disease | | | | | | |
| --- | --- | --- | --- | --- | --- | --- |
|  | **All-cause mortality** | | | | | |
|  | **White Cohort** | | **Black Cohort** | | **Asian Cohort** | |
|  | **Female** | **Male** | **Female** | **Male** | **Female** | **Male** |
| Events/total participants | 3,894/135,219 | 5,837/116,841 | 38/2,468 | 54/2,100 | 41/2,496 | 69/2,830 |
| NDI Quartile (Q) | HR (95% CI) | HR (95% CI) | HR (95% CI) | HR (95% CI) | HR (95% CI) | HR (95% CI) |
| Q1 Least deprived | **Referent** | | | | | |
| Q2 Somewhat deprived | 0.99 (0.90, 1.08) | 1.05 (0.97, 1.13) | 1.35 (0.25, 7.39) | 1.11 (0.10, 12.24) | 1.22 (0.39, 3.85) | 0.71 (0.30, 1.68) |
| Q3 Deprived | **1.10 (1.01, 1.21)*** | **1.16 (1.08, 1.25)***** | 0.63 (0.12, 3.23) | 2.27 (0.29, 17.77) | 0.78 (0.26, 2.34) | 0.57 (0.26, 1.27) |
| Q4 Most deprived | **1.38 (1.26, 1.51)***** | **1.59 (1.48, 1.71)***** | 0.79 (0.19, 3.33) | 2.22 (0.31, 16.13) | 1.21 (0.45, 3.23) | 0.80 (0.40, 1.58) |
|  | **Total cardiovascular disease incidence** | | | | | |
| Events/total participants | 28,005/135,219 | 34,057/116,841 | 667/2,468 | 575/2,100 | 586/2,496 | 858/2,830 |
| Q1 Least deprived | **Referent** | | | | | |
| Q2 Somewhat deprived | **1.04 (1.01, 1.07)*** | **1.04 (1.01, 1.07)*** | 0.87 (0.53, 1.42) | 1.26 (0.72, 2.19) | 1.23 (0.87, 1.73) | 0.93 (0.71, 1.21) |
| Q3 Deprived | **1.09 (1.05, 1.13)***** | **1.07 (1.04, 1.10)***** | 0.92 (0.60, 1.41) | 1.00 (0.60, 1.67) | **1.38 (1.02, 1.86)*** | 0.93 (0.73, 1.19) |
| Q4 Most deprived | **1.23 (1.19, 1.27)***** | **1.19 (1.16, 1.23)***** | 1.09 (0.74, 1.61) | 1.28 (0.80, 2.06) | **1.45 (1.09, 1.93)*** | 1.04 (0.83, 1.29) |
|  | **Ischemic heart disease incidence** | | | | | |
| Events/total participants | 4,685/153,046 | 9,705/134,312 | 82/2,955 | 88/2,486 | 147/2,878 | 354/3,315 |
| Q1 Least deprived | **Referent** | | | | | |
| Q2 Somewhat deprived | **1.12 (1.03, 1.21)**** | 0.99 (0.94, 1.05) | 0.39 (0.07, 2.36) | 2.02 (0.42, 9.71) | 0.59 (0.29, 1.20) | 0.90 (0.60, 1.35) |
| Q3 Deprived | **1.17 (1.07, 1.27)***** | 1.04 (0.98, 1.10) | 0.76 (0.21, 2.77) | 0.69 (0.14, 3.41) | 0.90 (0.51, 1.58) | 0.84 (0.58, 1.21) |
| Q4 Most deprived | **1.44 (1.33, 1.56)***** | **1.18 (1.12, 1.25)***** | 1.24 (0.39, 3.95) | 1.98 (0.49, 8.09) | 1.22 (0.73, 2.04) | 0.86 (0.62, 1.21) |
|  | **Cerebrovascular disease incidence** | | | | | |
| Events/total participants | 2,553/154,702 | 3,896/138,599 | 61/2,996 | 82/2,515 | 34/2,951 | 103/3,546 |
| Q1 Least deprived | **Referent** | | | | | |
| Q2 Somewhat deprived | 1.10 (0.98, 1.23) | **1.11 (1.02, 1.22)*** | 0.57 (0.12, 2.84) | 0.92 (0.15, 5.53) | 2.18 (0.42, 11.24) | 0.90 (0.37, 2.17) |
| Q3 Deprived | **1.19 (1.06, 1.33)**** | 1.09 (0.99, 1.20)^+^ | 0.76 (0.21, 2.76) | 1.85 (0.42, 8.07) | 1.56 (0.32, 7.49) | 1.06 (0.49, 2.29) |
| Q4 Most deprived | **1.47 (1.31, 1.64)***** | **1.45 (1.32, 1.58)***** | 0.81 (0.25, 2.60) | 1.89 (0.46, 7.68) | 2.92 (0.68, 12.48) | 1.53 (0.76, 3.09) |
| **Note**: Abbreviations: Hazard ratio: HR. CI: Confidence interval. Models adjusted for age. Significance: ***p<.001; **p<.01; *p<.05; and ^+^p<.1. **Boldface** indicates a significant association. | | | | | | |

| **Supplemental Table 9**. Longitudinal associations of deprived neighborhoods with all-cause mortality, incident total cardiovascular disease, ischemic heart disease, and cerebrovascular disease | | | | |
| --- | --- | --- | --- | --- |
|  | **All-cause mortality** | **Total CVD incidence** | **IHD incidence** | **CeVD incidence** |
| Events/total participants | 9,933/26,1954 | 64,748/261,954 | 15,061/298,992 | 6,729/305,309 |
| NDI Tertile (T) | HR (95% CI) | HR (95% CI) | HR (95% CI) | HR (95% CI) |
| T1 Least deprived | **Referent** | | | |
| T2 Somewhat deprived | 1.04 (0.96, 1.09) | **1.04 (1.02, 1.06)***** | **1.06 (1.02, 1.10)**** | **1.08 (1.01, 1.14)*** |
| T3 Most deprived | **1.27 (1.21, 1.33)***** | **1.10 (1.08, 1.12)***** | **1.12 (1.08, 1.17)***** | **1.26 (1.19, 1.34)***** |
| **Note**: Abbreviations: CVD, cardiovascular disease; IHD, ischemic heart disease; CeVD, cerebrovascular disease; Hazard ratio, HR; CI, Confidence interval. Models adjusted for age, sex, and ethnicity. Significance: ***p<.001; **p<.01; and *p<.05. **Boldface** indicates a significant association. | | | | |

| **Supplemental Table 10**. Sex-specific longitudinal associations of deprived neighborhoods with all-cause mortality, incident total cardiovascular disease, ischemic heart disease, and cerebrovascular disease | | |
| --- | --- | --- |
|  | **All-cause mortality** | |
|  | **Female** | **Male** |
| Events/total participants | 3,973/140,183 | 5,960/121,771 |
| NDI Tertile (T) | HR (95% CI) | HR (95% CI) |
| T1 Least deprived | **Referent** | |
| T2 Somewhat deprived | 1.00 (0.92, 1.08) | **1.07 (1.01, 1.14)*** |
| T3 Most deprived | **1.18 (1.09, 1.27)***** | **1.33 (1.25, 1.41)***** |
|  | **Total cardiovascular disease incidence** | |
| Events/total participants | 29,258/140,183 | 35,490/121,771 |
| T1 Least deprived | **Referent** | |
| T2 Somewhat deprived | **1.04 (1.01, 1.07)*** | **1.04 (1.01, 1.07)**** |
| T3 Most deprived | **1.11 (1.08, 1.14)***** | **1.10 (1.07, 1.13)***** |
|  | **Ischemic heart incidence** | |
| Events/total participants | 4,914/158,879 | 10,147/140,113 |
| T1 Least deprived | **Referent** | |
| T2 Somewhat deprived | **1.12 (1.04, 1.20)**** | 1.03 (0.98, 1.08) |
| T3 Most deprived | **1.22 (1.13, 1.31)***** | **1.08 (1.03, 1.13)**** |
|  | **Cerebrovascular disease incidence** | |
| Events/total participants | 2,648/160,649 | 4,081/144,660 |
| T1 Least deprived | **Referent** | |
| T2 Somewhat deprived | **1.10 (1.00, 1.22)*** | 1.06 (0.98, 1.15) |
| T3 Most deprived | **1.29 (1.17, 1.42)***** | **1.24 (1.15, 1.34)***** |
| **Note**: Abbreviations: Hazard ratio, HR; CI, Confidence interval. Models adjusted for age and ethnicity. Significance: ***p<.001; **p<.01; *p<.05; and ^+^p<.1. **Boldface** indicates a significant association. | | |

| **Supplemental Table 11.** Ethnic-specific longitudinal associations of deprived neighborhoods with all-cause mortality, incident total cardiovascular disease, ischemic heart disease, and cerebrovascular disease | | | |
| --- | --- | --- | --- |
|  | **All-cause mortality** | | |
|  | **White** | **Black** | **Asian** |
| Events/total participants | 9,731/252,060 | 92/4,568 | 110/5,326 |
| NDI Tertile (T) | HR (95% CI) | HR (95% CI) | HR (95% CI) |
| T1 Least deprived | **Referent** | | |
| T2 Somewhat deprived | 1.04 (0.99, 1.10) | 1.39 (0.46, 4.24) | 0.79 (0.45, 1.41) |
| T3 Most deprived | **1.27 (1.21, 1.34)***** | 1.21 (0.44, 3.32) | 0.82 (0.50, 1.33) |
|  | **Total cardiovascular disease incidence** | | |
| Events/total participants | 62,062/252,060 | 1,242/4,568 | 1,444/5,326 |
| T1 Least deprived | **Referent** | | |
| T2 Somewhat deprived | **1.04 (1.02, 1.06)***** | 1.04 (0.78, 1.39) | 1.05 (0.88, 1.24) |
| T3 Most deprived | **1.10 (1.08, 1.12)***** | 1.11 (0.86, 1.43) | 1.10 (0.95, 1.27) |
|  | **Ischemic heart incidence** | | |
| Events/total participants | 14,390/287,358 | 170/5,441 | 501/6,193 |
| T1 Least deprived | **Referent** | | |
| T2 Somewhat deprived | **1.06 (1.02, 1.10)**** | 0.81 (0.35, 1.89) | 0.99 (0.75, 1.30) |
| T3 Most deprived | **1.13 (1.08, 1.17)***** | 1.14 (0.56, 2.33) | 0.94 (0.73, 1.20) |
|  | **Cerebrovascular disease incidence** | | |
| Events/total participants | 6,449/293,301 | 143/5,511 | 137/6,497 |
| T1 Least deprived | **Referent** | | |
| T2 Somewhat deprived | **1.08 (1.02, 1.15)*** | 1.10 (0.43, 2.80) | 0.83 (0.46, 1.51) |
| T3 Most deprived | **1.26 (1.18, 1.34)***** | 1.30 (0.57, 2.97) | 1.29 (0.79, 2.12) |
| **Note**: Abbreviations: Hazard ratio: HR. CI: Confidence interval. Models adjusted for age and sex. Significance: ***p<.001; **p<.01; and *p<.05. **Boldface** indicates a significant association. | | | |

| **Supplemental Table 12.** Sex- and ethnic-specific longitudinal associations of deprived neighborhoods with all-cause mortality, incident total cardiovascular disease, ischemic heart disease, and cerebrovascular disease | | | | | | |
| --- | --- | --- | --- | --- | --- | --- |
|  | **All-cause mortality** | | | | | |
|  | **White Cohort** | | **Black Cohort** | | **Asian Cohort** | |
|  | **Female** | **Male** | **Female** | **Male** | **Female** | **Male** |
| Events/total participants | 3,894/135,219 | 5,837/116,841 | 38/2,468 | 54/2,100 | 41/2,496 | 69/2,830 |
| NDI Tertile (T) | HR (95% CI) | HR (95% CI) | HR (95% CI) | HR (95% CI) | HR (95% CI) | HR (95% CI) |
| T1 Least deprived | **Referent** | | | | | |
| T2 Somewhat deprived | 1.00 (0.93, 1.08) | **1.07 (1.00, 1.14)*** | 1.32 (0.27, 6.59) | 1.49 (0.32, 7.02) | 0.66 (0.24, 1.83) | 0.86 (0.43, 1.74) |
| T3 Most deprived | **1.18 (1.09, 1.28)***** | **1.34 (1.25, 1.42)***** | 1.01 (0.24, 4.26) | 1.37 (0.33, 5.68) | 0.95 (0.43, 2.12) | 0.74 (0.40, 1.38) |
|  | **Total cardiovascular disease incidence** | | | | | |
| Events/total participants | 28,005/135,219 | 34,057/116,841 | 667/2,468 | 575/2,100 | 586/2,496 | 858/2,830 |
| T1 Least deprived | **Referent** | | | | | |
| T2 Somewhat deprived | **1.04 (1.01, 1.07)*** | **1.04 (1.01, 1.07)**** | 1.06 (0.72, 1.56) | 1.01 (0.65, 1.57) | 1.13 (0.86, 1.47) | 0.99 (0.80, 1.24) |
| T3 Most deprived | **1.11 (1.07, 1.14)***** | **1.10 (1.07, 1.13)***** | 1.07 (0.77, 1.51) | 1.16 (0.78, 1.71) | 1.22 (0.96, 1.54)+ | 1.02 (0.84, 1.24) |
|  | **Ischemic heart disease incidence** | | | | | |
| Events/total participants | 4,685/153,046 | 9,705/134,312 | 82/2,955 | 88/2,486 | 147/2,878 | 354/3,315 |
| T1 Least deprived | **Referent** | | | | | |
| T2 Somewhat deprived | **1.13 (1.05, 1.21)**** | 1.03 (0.98, 1.08) | 0.81 (0.24, 2.72) | 0.80 (0.25, 2.61) | 0.84 (0.49, 1.45) | 1.03 (0.75, 1.43) |
| T3 Most deprived | **1.22 (1.13, 1.31)***** | **1.09 (1.03, 1.14)**** | 1.09 (0.40, 3.01) | 1.19 (0.43, 3.26) | 1.14 (0.72, 1.80) | 0.85 (0.64, 1.15) |
|  | **Cerebrovascular disease incidence** | | | | | |
| Events/total participants | 2,553/154,702 | 3,896/138,599 | 61/2,996 | 82/2,515 | 34/2,951 | 103/3,546 |
| T1 Least deprived | **Referent** | | | | | |
| T2 Somewhat deprived | **1.10 (1.00, 1.22)*** | 1.07 (0.99, 1.15) | 0.95 (0.25, 3.71) | 1.27 (0.35, 4.63) | 1.46 (0.44, 4.85) | 0.69 (0.34, 1.37) |
| T3 Most deprived | **1.29 (1.17, 1.42)***** | **1.24 (1.14, 1.34)***** | 1.06 (0.33, 3.43) | 1.53 (0.48, 4.89) | 1.59 (0.54, 4.63) | 1.21 (0.69, 2.11) |
| **Note**: Abbreviations: Hazard ratio: HR. CI: Confidence interval. Models adjusted for age. Significance: ***p<.001; **p<.01; *p<.05; and ^+^p<.1. **Boldface** indicates a significant association. | | | | | | |
